# Consumer Attitude Toward the Environmental Sustainability of Grain-Free Pet Foods

**DOI:** 10.3389/fvets.2018.00170

**Published:** 2018-09-24

**Authors:** Danielle M. P. Conway, Korinn E. Saker

**Affiliations:** Small Animal Clinical Science, College of Veterinary Medicine, North Carolina State University, Raleigh, NC, United States

**Keywords:** environment, sustainability, canine nutrition, grain-free, owner perception

## Abstract

This study investigated consumer knowledge and attitude toward environmental sustainability, grain-free diets (GFDs), and the influence of on-site environmental sustainability education on pet owner diet choices. A two-part questionnaire was designed, bracketing an educational brochure on environmental sustainability and GFDs. The study consisted of an informational brochure and two questionnaire sections, Q1 and Q2. Preliminary information regarding current diets, diet choice(s), views of environmental sustainability, the definition of GFDs, and the likelihood of feeding GFDs were gathered via Q1. Participants then read a factual brochure regarding pet food trends and environmental sustainability. After reading the brochure, participants completed Q2. Pet ownership of the survey population indicated 12/78 cared exclusively for at least one cat, 48/78 cared exclusively for at least one dog, and 18 cared exclusively for at least one dog and one cat. The majority (70/78) of survey responders fed a dry commercial product, 25/78 fed a canned commercial product, and 1/78 fed a commercial raw product. Prior to reading the brochure, 44.9% of participants were able to partially identify a GFD, 47.4% partially defined environmental sustainability, and 19.2% reported feeding a GFD. After reading the brochure, 67.6% of participants were able to identify a more environmentally sustainable diet vs. 55.9% prior to reading the brochure. A paired *T*-test demonstrated that after reading the brochure, people were significantly less likely to feed a GFD (*p* < 0.001). When participants already feeding a GFD were isolated, they demonstrated a higher likelihood to feed a GFD both before and after reading the pamphlet than the remaining population; however, the likelihood decreased from 8.4 ± 2.7 to 7.8 ± 2.7. The informational brochure was effective; participants were less likely to feed a GFD after reading the brochure. Although participants considered environmental sustainability important, factors independent of environmental sustainability influenced the likelihood of diet change. Participants already feeding a GFD also ranked environmental sustainability highly but were less likely to consider changing their pet's diet. These preliminary findings identify a need for public education regarding pet food choices that can have environmental consequences.

## Introduction

In recent decades, anthropomorphism of domestic animals has led to an increased inclusion into family units such that they are viewed as family members (1). A 2017 study by the American Pet Products Association found that approximately 107 million American households own a dog or cat, which has increased from 70 million in 2012 (2, 3). The pet product industry and pet food manufacturers have responded to this culture shift with trendy diets that questionably align with animal health, nutritional biochemistry, and physiology (4, 5). These diet trends include grain free, homemade and utilization of “human grade” ingredients. Likely the most well-known and best advertised among these are the grain-free diet (GFD) trend in which pet food companies market these diets as high protein “ancestral diets” that are healthier than their grain inclusive counterparts. These trends challenge the concept of environmental sustainability (ES) by promoting excessive intake of animal based protein feed ingredients, by the over use of “human grade” ingredients that compete directly with the human food system and by discouraging the use of more sustainable ingredients in pet food such as grains and by-products (6). These factors can all be quite impactful on the environment as supported by Okin et al. who reported that dog and cat animal product consumption is responsible for the release of up to 64 ± 16 million tons of CO_2_-equivalent methane and nitrous oxide, two powerful greenhouse gasses (7).

The overall premium pet food marketplace is estimated at 18 billion dollars (8). While reviewed and discussed by the pet food industry, objective statistics are not readily available. The Pet Food Industry group suggests grain free pet food sales in 2015 were 2.6 billion dollars with a growth rate of 25% (9), and the GfK marketing group report similar statistics with grain free sales at 2.7 billion dollars annually in the US, comprising 29% of the pet food market. Currently, the top selling dry dog food on both of the top grossing pet food purchase websites, chewy.com and amazon.com, is a grain free pet food (Amazon Top Selling Dry Dog Food, Chewy Top Selling Dry Dog Food). Together, these data suggest that the grain free pet food sector is growing and represents an increasingly substantial portion of the overall pet food marketplace. In spite of growing popularity there is no scientific data to support that grain free feeding is healthier than grain inclusive feeding. On the contrary grains are included in pet foods to supply a highly digestible and efficient source of glucose, amino acids, fiber, phytonutrients and micro minerals (U.S. Department of Health and Human Services and U.S. Department of Agriculture. Dietary Guidelines for Americans. 8th Edition). Grain free diets have a more taxing environmental impact than their grain inclusive counterparts because they are advertised to contain a high percentage of animal based protein sources, utilize only “human grade” ingredients, and discourage the use of by-products and gains. Several studies have demonstrated that the overall environmental impact of animal based proteins is higher than plant based protein sources. A 2013 study by Swanson et al points out that the energy input to protein output of animal based proteins at 25:1 is 11 times higher than that of plant based proteins (2.2:1) (6, 10). While there are no rigorously reviewed objective statistics available with regard to human grade ingredients and by-products, it is a reasonable conclusion that utilization of ingredients not in direct competition with the human food system, and maximize efficient use of environmentally taxing animal ingredients not desirable as foods in the Western market, would make for an efficient and nutritious environmentally sustainable pet food.

Additionally, companion animals are increasingly recognized as having a positive impact on human health (11). As human populations increase, however, companion animal nutrition should be considered with the same goals applied to meeting human nutritional needs in terms of balancing resources to achieve ES. Consumer knowledgeability of pet food diets and their related ES is presently not well understood and needs to be studied if long-term nutrient product and ES balance is to be achieved (6, 7). Here, we set out to characterize consumer knowledge of GFDs and their related ES, since these diets are among the most commonly encountered non-traditional companion animal diets. Specifically, we used a questionnaire-based approach to evaluate: participant pet owners presumed of ES; their knowledge of GFDs and ES; and if when presented with an informational brochure objectively linking GFD feeding to decreased ES, that information would influence them away from GFDs.

## Materials and methods

Participants were asked to fill out an initial questionnaire (Q1) (Image 1), read an educational brochure (Image 2), then fill out a second questionnaire (Q2) (Image 1); all of which were written for this study and are shown in Supplementary Material. The brochure was a tri-fold design, including short, concise factual points on basic dog gastrointestinal physiology, grain facts, ancestral diet trends, by-product definition and examples, sustainability facts including an example of the environmental cost of feeding pets an animal- vs. animal and plant-based diet, and finally guidelines for a nutritionally sustainable pet food. A pilot with 56 participants was utilized to edit the flow and language of the questionnaires and research material (not shown). The study reported here included 78 participants from the North Carolina State University College of Veterinary Medicine (NCSU-CVM), a local general small animal veterinary practice, a local pet products store, and visitors to an NCSU-CVM Open House. All data were collected voluntarily, within a 3-month time span, anonymously, and participants were not compensated. The NC-State IRB was consulted and additional approval deemed not necessary. Data collection was broken into two sections; the first was to collect general information such as the quantity of dogs and cats in the home, type and brand of current food, willingness to feed a GFD, importance of ES, and criteria for selection of a pet food was gathered via Q1. Q1 also tested the participants' ability to correctly identify the definitions of GFD and ES and their ability to select the most ES feeding option for their pet. After participants read the brochure, the second half of data collection (Q2) examined participant impressions, likelihood to change diet based upon information learned and why, likelihood to feed a GFD, and selection of the most ES feeding option for pets. Several questions were replicated in Q2 from Q1 to determine the participants' ability to comprehend and gain knowledge from the brochure. Participants were given space to fill out the questionaries' away from the researcher and the Researcher did not help with any questions to avoid biasing the participants.

Quantitative assessments of responses were summarized to highlight consumer knowledge base and diet preferences. Due to the exploratory nature of the study, similar studies were not available so a cut-off value for importance was set at >5. Comparisons between selected questions on Q1 vs. Q2 were made utilizing a variety of statistical analyses, such as paired, Wilcoxon two-sample *T*-tests, and chi-square test for homogeneity.

## Results

### Questionnaire 1 (Q1) before reading brochure

Pet ownership of the survey population indicated 12/78 cared exclusively for at least one cat, 48/78 cared exclusively for at least one dog, and 18 cared exclusively for at least one dog and one cat. The majority (70/78) of survey responders reported feeding (nonexclusively) a dry commercial product, 25/78 fed a canned commercial product, 1/78 fed a commercial raw product. 10/78 participants fed a non-commercial product, either home prepared, raw, or cooked. The percentage of responders that indicated they were currently feeding Grain-free, Natural, Organic, or Human Grade were 19.2, 15.4, 5.1, 2.6%, respectively. When asked what factors influenced their selection of food for their pet, 60.3% chose ingredient list as the most influential factor; 55.1% selecting cost, 53.9% selecting company reputation, 46.2% choose veterinary recommendation; and 41% indicated ease of purchasing as the most influential factor. Least important factors influencing pet food selection were packaging (2.6%), environmental sustainability (14.1%), and both a GFD and a diet that is free of corn wheat and soy (16.7%).

Participants were asked to describe a GFD by identifying in the questionnaire which terms they would commonly associate with carbohydrates and grain. Likewise, they were given the option of “unsure.” Nearly 70% of responders correctly identified a GFD would be free of wheat, soy, and/or corn, while 96% identified a GFD to be free of barley. Although able to identify the absence of specific grains as being part of a GFD, 35% of these participants chose inappropriate defining terms for GFD as well. A small number of responders (20.5%) indicated they were “unsure” of the definition of GF. While only one responder was able to correctly describe a GFD based on available terms/terminology provided. Incorrect GFD descriptors commonly chosen included: diet free of “fillers” (63%) and diet free of carbohydrates (10%).

Participants were asked to indicate their understanding (belief of) of truisms regarding ES. Six options were provided with a seventh as “unsure.” Three of the six possible responses were true statements regarding ES, the remaining 3 were incorrect statements. While 14% of participants were able to correctly identify the ES truisms, 20% identified at least 1 or 2 of the 3 truisms, and 41% of participants chose at least one of three incorrect answers. Amongst this group, 89% selected at least one correct answer. Additionally, participants were asked to score, on a sliding scale from not important (0) to important (10), how important ES was to them. The average score was 6.9 ± 2.4, with 77% scoring a 5 or greater.

### Questionnaire 2 (Q2) after reading brochure

Clinical experience suggests some pet owners may hold strong opinions about their choice of pet food and be less willing to re-evaluate that choice when presented with new information. To screen for this effect, participants were asked to select all options (anger, positive, negative, indifferent, informative, or enlightening) that apply regarding their first impression of reading the brochure. A positive response reported by 76/78 (97.4%) participants was indicated by selecting at least one of the descriptors: informative or enlightening. Approximately 2.6% of participants indicted a negative reaction to the brochure, and 6/78 (7.7%) selected an indifferent first impression.

We next investigated whether there was any relationship between the participants understanding of and feelings regarding ES and diet, specifically GFDs, by evaluating responses to various pre- and post-brochure questions. To determine whether a correlation existed between the responders perceived importance of ES and their willingness to switch away from a less ES GFD should they discover GFD were less ES, we compared the responses to these two ES-associated questions in Q1. Participants ranked how important ES was to them on a sliding scale from not important (0) to important (10), and the average score was 6.9 ± 2.4, with 77% scoring a 5 or greater. Individuals who indicated they would be willing to switch away from a GFD in order to be more ES were found to score ES higher (7.3 ± 2.0, *p* < 0.032) than those who were not (5.9 ± 2.5).

**Figure 1:**
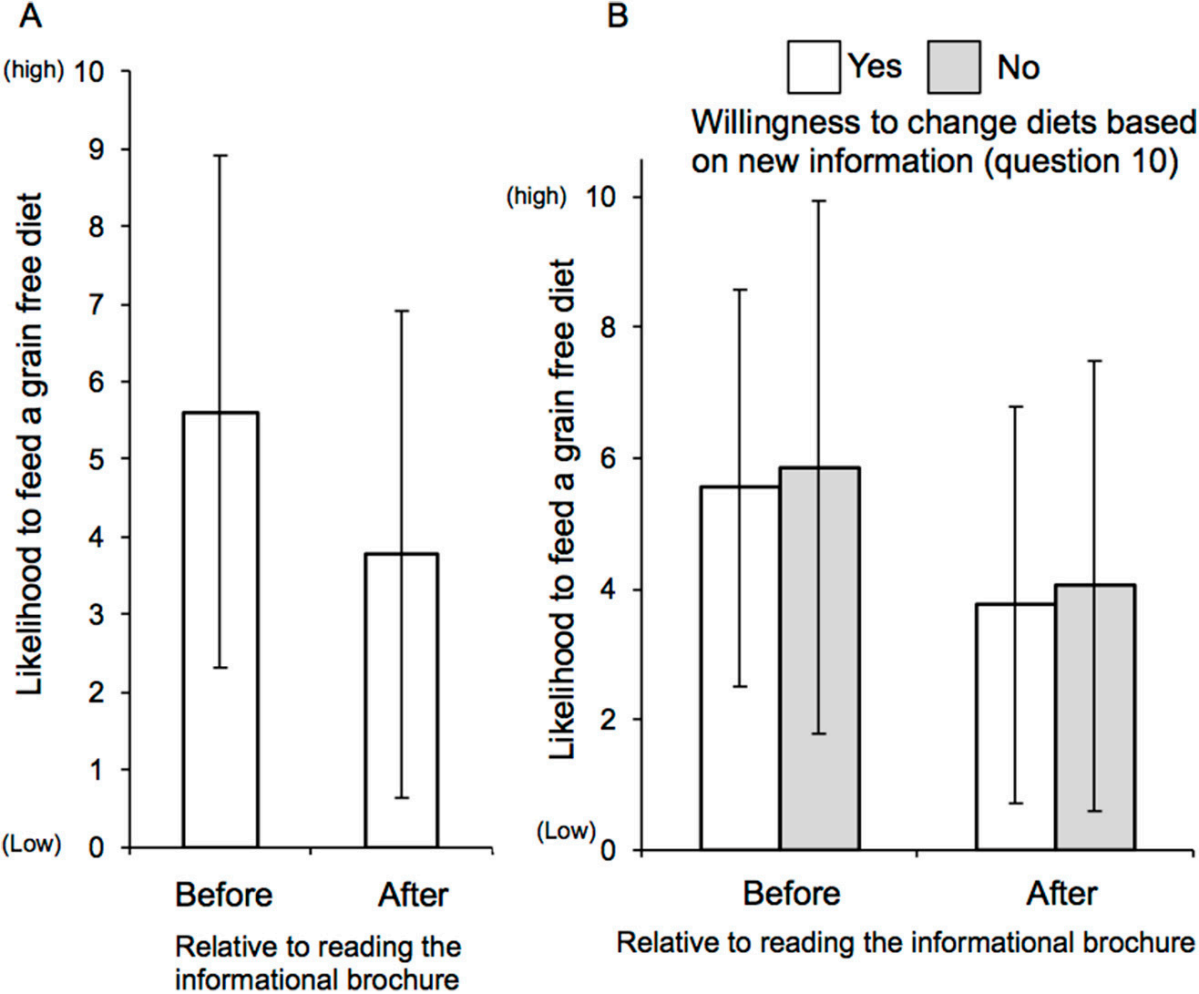
Participant likelihood to feed a grain-free diet before and after reading the pamphlet. Likelihood to consider a GFD before and after brochure education. **(A)** Participants were asked to score (0 = not likely, to 10 = likely) whether they would consider feeding a GFD before and after reading the brochure detailing the lower ES of GFDs. Before compared to after *p* = 0.003. *n* = 74. (8) Likelihood to consider a GFD before and after brochure education in combination with willingness to change diet to be more ES. The responses to **(B)** were further segregated by a “yes” or “no” answer to whether participants would consider changing the ir diet if new information were presented linking lower ES to their current diet choice. Before compared to after segregated by willing to change diet based upon ES *p* = 0.194, and before compared to after segregated by not willing to change diet based upon ES *p* = 0.008, *n* = 73.

We measured whether reading the informational brochure persuaded some to consider not feeding a GFD. Participants were, on average, less likely to consider a GFD after reading the pamphlet (Figure 1). As expected, those indicating a willingness to use new information to be more ES were less likely to feed a GFD after the brochure than before, but the same trend was also observed for those indicating an unwillingness to use new information showing GFDs to be less ES when choosing a pet food (Figure 1).

When the participants were asked if they would actually change, not just a willingness to consider GFDs, their pets current diet based on the brochure information, 65.3% answered 5 or less (out of 10), an average of 3.8 ± 3.1. Reasons provided as to why they were less willing to change included: “currently feeding a diet that has grain” (43.6%), “their veterinarian recommends the current diet” (24.4%), or “their animal is doing well on the current diet” (66.7%). For those who selected, “their animal is doing well on the current diet,” and did not report feeding a GFD (53.8%), their average pre-brochure (Q1) likelihood to consider a GFD was on average 4.5 ± 2.8 out of 10, which decreased to 2.7 ± 2.4 (Figure 1) after reading the brochure (Q2). Conversely the same population of participants indicated a low (3.5 ± 2.8 out of 10) willingness to actually change diets. This group also ranked ES importance as an average of 6.9 ± 2.3 out of 10.

**Figure 2:**
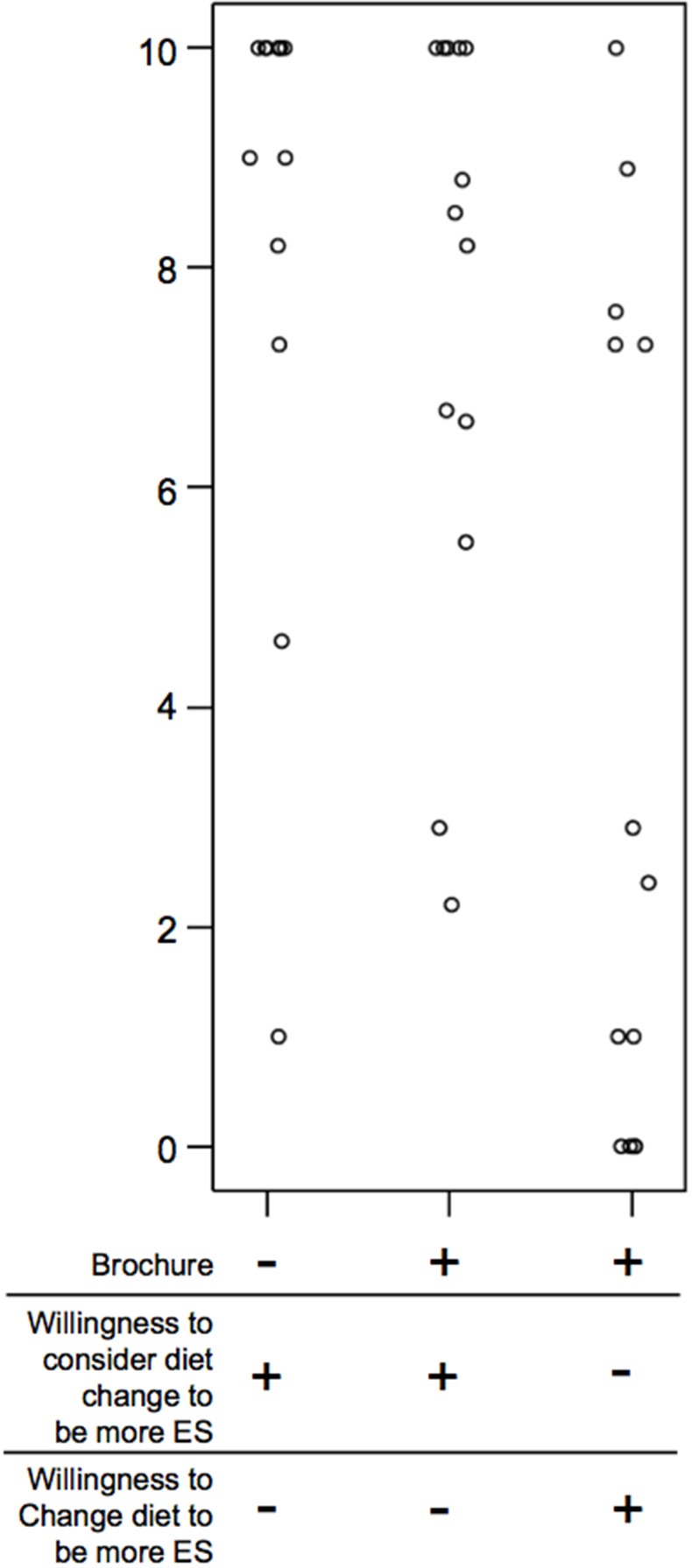
Participant willingness to change diet to be more environmentally sustainable before and after reading the pamphlet. Likelihood to consider, and change a GFD before and after brochure education on the ES of GFDs by participants who identified as already feeding a GFD at time of study. Participants who self-identified as feeding a GFD were asked to score (0 = not likely, 10 = likely) their likelihood to consider feeding a GFD before, and after brochure education on the ES of GFDs. The group is further segregated by whether they would change diet to in response to new information illustrating the ES of a GFD. *n* = 13.

Those participants whom self-identified as currently feeding a GFD on Q1 (19.2%), indicated a high tendency (8.6 ± 2.8) to consider a GFD before brochure education (Q1), which was much higher than the remainder of the group's average of 5.0 ± 3.1 (*p* < 0.001). Unlike the remainder of the cohort who tended to be less likely to consider a GFD after brochure education (Q2), the GFD group remained likely to consider a GFD with an average score of 7.6 ± 2.7 (Figure 2) compared to 2.8 ± 2.4 for the remainder of the group. Interestingly, when the GFD self-identified group was asked to score how likely they were to actually change their diet, the group segregated into two distinct populations: one likely to change, as indicated by a high score, and one not (Figure 2). Given that ES was ranked high in the GFD feeding population (7.7 ± 1.6 out of 10), some change was expected, but the two populations suggests another element may be influencing decision making such as strongly held beliefs about what constitutes a healthy pet food and possibly some degree of cognitive dissonance.

**Figure 3:**
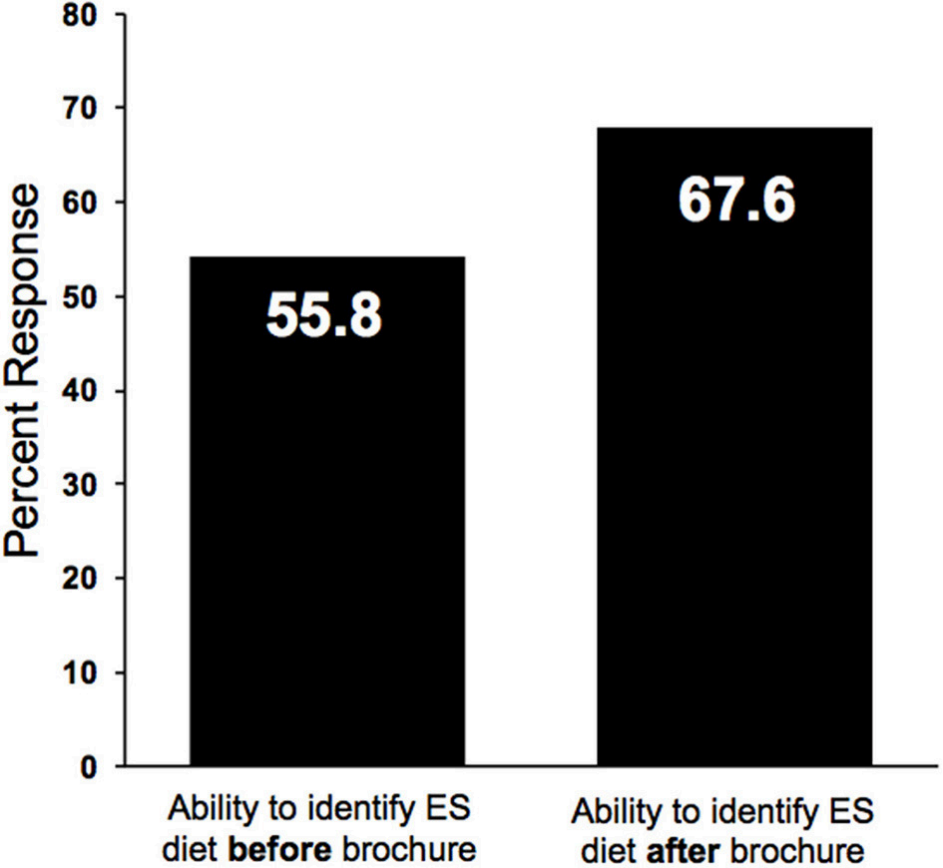
Participant ability to correctly identify the most ES diet before and after reading brochure. Participants were asked to identify the most ES diet before and after reading brochure. Percentages represent the part of the study population who were able to correctly identify the most ES diet. *n* = 68.

We examined whether ES and GFD education through the informational pamphlet could be assimilated to identify the most ES diet from a selection of diets. Figure 3 demonstrated that prior to reading the pamphlet, 55.8% of participants recognized that a commercial pet food containing moderate levels of chicken and rice as a more sustainable protein source than beef (homemade or commercial), and after, 67.6% were able to make this identification.

To evaluate whether demographic traits of the participant population influenced questionnaire results, the participant population was categorized into three groups based on location and then on participant education attainment. Location groups included: veterinary teaching hospital (VTH) clients, local referral clinic clients, local pet product store, and veterinary college Open House participants. Levels of education included: high school or equivalent, post high school (associate, bachelor, or specialized trade degree/certification), graduate degree, human medical, and animal medical degrees. A Chi-squared test for homogeneity of proportions found differences in participants' ability to define GFDs and ES. The VTH clients were better able to define GF (*p* = 0.012) and ES (*p* = 0.0624) when compared to the other two location groups. Participants with a higher education level were better able to define GF (*p* = 0.02); however, there was no correlation between education level and ability to define ES (*p* = 0.21). Education level did not influence participants' likelihood to feed GF (*p* = 0.97) or their ranking of ES (*p* = 0.316).

## Discussion

Feeding based on dietary trends that are in direct competition with environmental sustainability such as human grade, natural, ancestral, raw, homemade, and grain-free have become increasingly popular for reasons not well researched in peer reviewed literature. According to a 2015 study by Kumcu and Woolverton, the desire to feed pets like members of the family and belief that these trends are the best for their pets are two hypothesizes driving these trends despite lack of scientific evidence to back up their popularity. While GFD sales slowed from a 42% growth rate in 2013 to a 24% growth rate in 2014, they still accounted for 30% of total pet food sales in the United States (9). Clinical experience has shown that while the GFD trend is popular and owners will present to veterinary nutrition services requesting a diet free of grains for a variety of perceived reasons, many owners may be less sure of the factual benefits and/or consequences of feeding a GFD to their pet. The purpose of this study was to evaluate participant (pet owner) knowledge and attitude toward environmental sustainability, its relation to GFDs, and to determine the influence on-site GFD and ES education would have on influencing pet owner diet choices. Specifically, if participants would be less likely to feed a GFD after reading an informational brochure.

General information collected from the questionnaire revealed that the majority of participants were dog owners who fed a dry commercial product. This appears to be in line with the percentage of dry (kibble) dog food sales across the United States (12). Top reasons for their choice of diet included that they were looking for a selected ingredient(s) in the diet, cost of the food, pet food company reputation, and a recommendation by their pet's veterinarian. This implies that veterinarians in general practice can be very influential in the diet choice(s) their clients make for their pets. Study participants identified packaging and environmental sustainability as the least important factors influencing their pet food selection. This suggests that how the product is presented or marketed is of lesser interest than the company that markets the product. This is interesting as pet food companies invest heavily in how to “dress” the product to attract sales. The fact that the diet was GF and/or containing corn, wheat, and soy raises the question of which ingredients (identified as a top reason for diet selection) pet owners are concerned about most.

Accurate identification of GFD ingredients was not common, and based on responses, participants demonstrated confusion between frequent ingredient list words. Surprisingly, only one individual in the study population selected both correct terms to define a GFD.

There appeared to be misperception of which foodstuffs are defined as carbohydrates and that other starches, vegetables, and legumes replace grains in a GFD as the carbohydrate source. Our study results reflect this misperception as the potato option was rarely identified as being part of a GFD, while barley, corn, soy and wheat were nearly always identified to be absent in a GFD. This suggests lack of clarity of our study participants regarding the relationship between GF and carbohydrates. Although not part of Q1 or Q2, a pet owners' perception of what a dog should eat may stem from the ancestral diet trend. The notion that dogs should be eating like their wolf ancestors, assumedly an all-meat type diet devoid of carbohydrates, may be a factor in owners understanding of the role of dietary carbohydrates in canine nutrition (4, 13–15). This could possibly influence their ability to identify vegetables as common carbohydrate sources in canine GDFs.

Predominant GFD descriptor selection by participants was “diets free of fillers and by-products.” This level of selection suggests participants associate the word “fillers” with grain, thus driving high selection. Although lacking a clear definition, “fillers” have been marketed as a low cost ingredient added to provide dietary fiber, bulk, or some other non-nutritive purpose in commercial pet foods in order to bolster profit margins. In commercial pet foods, grains, and certain cereal grain derivatives (e.g., corn gluten meal, soybean germ, corn oil) are included to provide nutritive value as an excellent source of carbohydrate (calories, glucose), protein (amino acids), and fat (calories, essential fatty acids). A very small portion of the cereal grain (i.e., wheat gluten) would be added for its functional value (e.g., texture enhancer). The term “by-product” has long been a point of controversy between pet owners, veterinarians, and nutritionists. The Association of American Feed Control Officials (AAFCO) defines by-products as “*Secondary products produced in addition to the principal product*” (16). Based on this definition, any animal product, secondary to skeletal meat, obtained from a slaughtered carcass would be a by-product. Liver and other organs are considered by-products, just as chicken by-product or meat and bone meal are considered common animal by-products. The nutritive value of animal by-products is generally significantly higher than the skeletal meat alone. Additionally, by-products are part of the prey animal predators consume first (e.g., wolves, feral dogs) (17). Many chew treats are “by-products,” including rawhides, bully sticks, and pig ears. A ES consideration is that many animal “by-products” are not in direct competition with the human food supply, at least in the US. Perhaps then our study participants are lacking a clear definition of both “fillers” and “by-products,” and thus commonly consider both as cheap ingredients that lacks nutritive value. These observations exemplify one theme that emerged from these data: the potential to confuse, or conflate, the definition of certain ingredients, ultimately hindering participant's ability to describe a GFD.

A *sustainable food system* meets the nutritional needs of an individual without compromising the ability of future generations to meet their nutritional needs (6). While animal and crop production focuses on meeting the nutritional needs of individuals, it concurrently has a quantitative “footprint” on the environment (10). This footprint is measured by physical space, energy use, gas waste production (e.g., carbon dioxide and methane), and water use (6). Animal production has a measurably greater environmental footprint vs. crop (corn, barley, rye) production (18, 19). While participants ranked ES with high importance (77% scoring greater than 5), they apparently struggled with the definition of environmental sustainability. Over half (59–63%) were unable to identify at least one truism regarding the definition of ES. This suggests that although owners rank ES as important, their understanding of ES is not widespread, and possibly confounded by words like “organic,” “natural,” and “holistic” in pet food labeling.

Participant willingness to consider a grain free diet was measured before and after reading an informational brochure. It provided facts that indicate feeding GFDs has a more substantial adverse impact on ES than feeding a grain-inclusive diet. This is not to imply that a pet diet needs to be devoid of appropriate animal protein sources, but the choice of animal protein (e.g., chicken vs. beef) and the concentration of animal protein ingredients in a diet can strongly influence the footprint in a positive or negative direction. Reading through the brochure appeared to be a positive, informative, enlightening experience for most participants (97.2%), prompting some to more strongly consider ES when feeding their dog.

Although a high percentage of participants indicated ES was important to them, the actual likelihood of the ES-concerned population to change their pet's diet was low. There may be several reasons for this dichotomy. Prior to reviewing the educational brochure, these pet owners indicated factors that strongly influence their pets' food selection. Cost, ingredients, and veterinary recommendation far outweighed ES, perhaps because these factors are more tangible than ES to most individuals. Specifically targeting ES education to veterinary practitioners may be warranted, as they appear to be quite influential in diet selection by pet owners. An ES diet will likely be more economical to feed compared to a GFD and can be equally as nutritionally appropriate for the pet as can be determined by the nutritional adequacy claim on the diet label. A second reason for the dichotomy may be that the majority of our population was already feeding a grain-inclusive diet. The sub-population of participants feeding a GFD (19.2%), in contrast, were much less likely to consider a non-GFD, and were less likely to consider changing even though this group also rated ES importance highly.

The VTH study participant population were able to more correctly define both GF and ES as compared to the participant population identified at local referral clinics, local pet product stores, and those identified at the veterinary college community Open House event. Further, participants with a higher education level were better able to define GF (*p* = 0.02). The assumption drawn from these data might suggest that socio-economic factors were influential in our study results. Interestingly though, there was no correlation between education level and ability to define ES, and education level did not influence participants' likelihood to feed GF or their ranking of ES as selection criteria of diet selection for their pet. Together, these data suggest pet owners may be poorly educated on what constitutes a GFD and on ES in general. Furthermore, making future diet choices keeping in mind ES may be helped by directed education in certain populations, but that established behaviors in pet feeding may be more resistant to change.

Study limitations that may have possibly influenced responses to Q1 and Q2 would be the brochure was only available in English. There is a highly diverse population of pet caregivers in this area of the state with varying levels of English comprehension, reading, speaking, and writing. Ensuring the study materials were comprehensible to every study participant could likely have resulted in different responses. Ensuring that every possible response to a question in Q1 and Q2 was un-ambiguous to limit any confusion or incorrect assumptions may have influenced data results. Whether this would have changed the study conclusions is not known, although an attempt was made to prevent these potential limitations by running the pilot-study involving 56 individuals.

The informational brochure appeared to be an effective tool to educate participants. Participants were less likely to consider feeding a GFD after reading the brochure and improved in their ability to identify an ES diet. Although participants considered ES important, factors independent of ES influenced the likelihood of diet change. The data suggests that consumers overall have a poor grasp of the concept of GF feeding and are impressionable to advertising and recommendations made by a variety of sources. This study indicates that there is potential for providing accurate information to consumers via discussion, brochures, or television and radio commercials. The data supports that consumers value ES and are open to education regarding the ES of pet foods. Commercial pet food companies not previously offering a GFD have also begun to manufacture them, presumably to capitalize on this market. Given the results of this study, veterinarians and other animal health professionals are likely to encounter increased confusion regarding GFDs and should be ready with basic facts to help owners navigate these diets.

## Author contributions

DC contributed via generation of study idea, design, data collection, data interpretation, and manuscript preparation. KS contributed to study design, data interpretation and manuscript preparation.

### Conflict of interest statement

The authors declare that the research was conducted in the absence of any commercial or financial relationships that could be construed as a potential conflict of interest.
