# Supplementary material for: Consumer Attitude Toward the Environmental Sustainability of Grain-Free Pet Foods
Source: Front Vet Sci. 2018 Sep 24;5:170. doi: 10.3389/fvets.2018.00170 (PMC6166590; doi:10.3389/fvets.2018.00170)
Supplement: Supplementary file 1 [file Image_1.PDF]

Consumer attitude toward environmental sustainability and current pet food trends

This questionnaire is authored by Danielle Conway, a small animal intern at NCSU-CVM as part of a research requirement for the internship (nutrition-focus) training program. It is not sponsored by a pet food company.

Part One: please answer these questions PRIOR to reading the accompanying brochure

1. Do you have a dog(s), cat(s) or both? *Circle all that apply:*
   Dog: 1 2 3 4 or more
   Cat: 1 2 3 4 or more

2. What do you feed your pet? *Circle all that apply*:

Commercial product(s):	Noncommercial product(s)
a. Canned<br>b. Dry<br>c. Raw (frozen or freeze dried)<br>d. Grain free<br>e. Natural<br>f. Organic<br>g. Human grade<br>h. Unsure	a. Home-prepared<br>b. Raw (meat, vegetables, dairy)<br>c. Cooked (meat, vegetables, dairy)<br>d. Human Grade

3. If you feed your pet a commercial product please list the food brand and type
   (ex: Wellness Senior Fish & Potato, canned food)
   ____

4. Please *circle* all options that you feel describe a **grain-free** diet

   a. A diet free of fiber
   b. A diet free of "fillers"
   c. A diet free of carbohydrates
   d. A diet that contains potato
   e. A diet that contains barley
   f. A diet free of "by-products"
   g. A diet free of wheat, soy and/or corn
   h. unsure

5. How likely are you to consider a **grain-free** feeding approach for your pet? *Please make a dot/dash to indicate your choice:*

   ____
   Not likely **Very likely**

6. *Please circle* all of the following statements you feel are true about **environmental sustainability**:

    a. Organic is more environmentally sustainable than conventional
    b. A natural/holistic option is more sustainable than a conventional one
    c. Is defined as the preservation of resources for future generations
    d. Is defined as a method of harvesting or using a resource so that the resource is not depleted or permanently damaged
    e. Energy conservation and energy efficiency are the same as they apply to environmental sustainability
    f. Protein sources are variable in their environmental sustainability scoring (ex. egg is a more sustainable protein option than beef)
    g. Unsure at this time

7. On a sliding scale, how important is **environmental sustainability** to you?
   Please make a dot/dash to indicate your choice:

   Not important **Very important**

8. Please *circle* the pet diet that you believe to be the **most** environmentally sustainable:

    a. A homemade grain-free diet containing high levels of beef
    b. A commercial grain-free pet food containing high levels of beef
    c. A homemade diet containing moderate levels of chicken and rice
    d. A commercial pet food containing moderate levels of chicken and rice

9. Please *circle* all factors that influence your selection of a pet food:
   Please indicate your top three criteria with a star (*).

    a. Cost
    b. Recall history
    c. Company reputation
    d. Ingredient list
    e. Natural ingredients / Organic ingredients
    f. Packaging
    g. Grain-free
    h. Free of corn, wheat and soy
    i. Protein source (ex. chicken, beef, fish, etc)
    j. Protein content (ex. restricted , moderate, enriched)
    k. Environmental sustainability
    l. Recommendation by my veterinarian
    m. The diet is backed by scientific research
    n. Ease of purchase (ex. sold at a grocery store or large pet store or boutique or veterinary office)

10. If you were to discover that grain-free diets have a **more negative** environmental impact than diets that contain grain(s); would that information play a role in your decision to select a diet for your pet? Please circle one.

    Yes **No**

Please read the accompanying brochure on *Environmental Sustainability* before proceeding with the <u>Part Two</u> of the questionnaire:

<u>Part Two:</u>

11. Please *circle* all that apply regarding your first impression of the pamphlet you just read:

    a. Anger
    b. Positive
    c. Negative
    d. Indifferent
    e. Informative
    f. Enlightening

12. How likely are you to consider changing the diet you are currently feeding based upon the information provided in the pamphlet?
    Please make a dot/dash to indicate your choice

    Not likely ________________________________ **Very likely**

13. Please *circle* all statements that apply regarding your response to question #12:

    a. My pet is doing well on their current diet
    b. My veterinarian recommended the current diet
    c. I am already feeding a diet containing grain
    d. I believe grain-free is the best diet choice for my pet
    e. Environmental impact **does not** factor into my pet food decision
    f. Other: ____

14. After reading the pamphlet, how likely are you to consider a grain-free feeding approach for your pet? *Please make a dot/dash to indicate your choice:*

    Not likely ________________________________ **Very likely**

15. Please *circle* all statements that apply regarding your response to question #14:

    a. My pet is doing well on their current diet
    b. My veterinarian recommended the diet
    c. Cost factors into my pet food decision/ purchase
    d. I believe grains provide nutritional benefit to pets
    e. Environmental impact **does** factor into my pet food decision
    f. I believe grain-free is the best diet choice for my pet
    g. Other: ____

16. Please *circle* the pet diet that you believe is the most environmentally sustainable:

    a. A homemade grain-free pet diet containing high levels of beef
    b. A commercial grain-free pet food containing high levels of beef
    c. A homemade grain-free pet diet containing moderate levels of chicken and rice
    d. A commercial pet food containing moderate levels of chicken and rice

Optional: Please *circle the correct response for questions 17-20.*

17. How likely are you to purchase a new collar for your pet?
    Please make a dot/dash to indicate your choice

    Not likely ______________________________ **Very likely**

18. Are you
    a. Male
    b. Female
    c. Other

19. What is your age?
    a. 18-21 years
    b. 22-30 years
    c. 31-49 years
    d. 50-70 years
    e. >70 years

20. What is your level of education:

    a. High school or equivalent
    b. Post high school (associate, bachelor degree, trade)
    c. Graduate (Masters, PhD)
    d. Human medical (MD, PA, RN)
    e. Animal medical (DVM, VMD, RVT)
