# Supplementary material for: Consumer Attitude Toward the Environmental Sustainability of Grain-Free Pet Foods
Source: Front Vet Sci. 2018 Sep 24;5:170. doi: 10.3389/fvets.2018.00170 (PMC6166590; doi:10.3389/fvets.2018.00170)

Environmental sustainability and current pet food trends

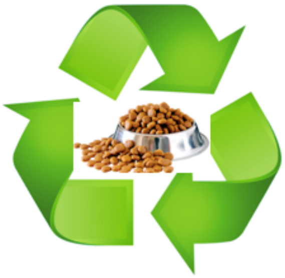

Many pet food companies are promoting trends that are in direct conflict with environmental sustainability.

This brochure will take a closer look at these current trends, how they affect environmental sustainability and how we can better meet the nutritional demands of growing human and animal populations.

This pamphlet was created by The Small Animal Clinical Nutrition service at NCSU College of Veterinary Medicine © It is not sponsored by a pet food corporation.

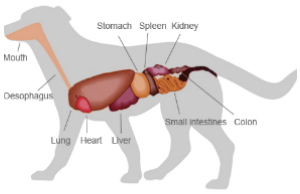

<u>Physiology Facts:</u>

1. Dogs & cats have digestive enzymes that allow efficient utilization of carbohydrate grains for daily energy/calories.

2. Ground and cooked (processed) grains can be >90% digestible.

3. There is no scientific evidence to support that corn or other grains cause health problems in animals.

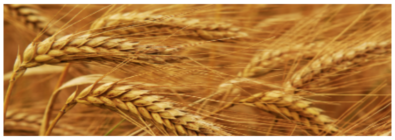

Grain Facts:

1. Grains are:
   - **rich in B vitamins**
   - **a good energy-calorie source**
   - **a good fiber source to help promote solid stool & feed healthy gut microbes**

2. Research in people links increased wheat and rye intake with a lower incidence of colon cancer

3. Many grain-free foods have comparable carbohydrates levels to their grain-inclusive counterparts
   - Grain-free does not mean carbohydrate free.
   - **Grain free foods swap out grains for alternative carbohydrate sources such as potato and pea.**
   - White potato (a common carbohydrate source in many grain-free diets) is high on the glycemic index.

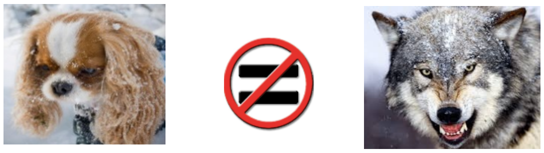

Ancestral Diet Trend:

1. New research shows that dogs & wolves vary genetically in several ways.
   - **A 2013 study determined that dogs possess more genes with key roles in starch digestion than wolves.**
   - **Domesticated animals have significantly longer life spans as compared to wolves & other wild animals largely in part due to advancements made in veterinary medicine and domestic animal nutrition.**

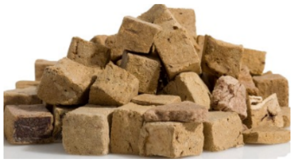

By Products:

1. By definition, by-products…
   - **Include lung, spleen, kidney, brain, liver, blood, bone, stomach and intestine.**
   - **Do NOT include hair, horns, teeth, and hooves.**
   - **Are not made from euthanized animals or road kill.**

2. By-products are …
   - **An excellent source of nutrients.**
   - **The part of the prey animal predators consume first.**
   - **Are not in direct competition with the human food supply.**
   - **Most chew treats are by-products including rawhides, bully-sticks, pig ears, etc.**

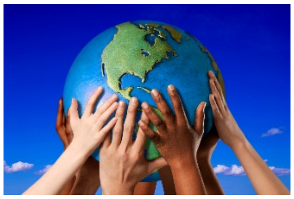

Sustainability Facts:

1. A ***sustainable food system*** meets the nutritional needs of an individual without compromising the ability of future generations to meet their nutritional needs.
   - ***Will we have enough (food) to go around?***

2. ***Footprint*** = a measure of demand upon the earth. It is a measure of ***sustainability***.
   - **Footprint can be measured by: physical space, energy use, greenhouse gas waste production (carbon dioxide, methane) and water use.**

3. Footprint of pet food ingredients:
   - **Chickens and turkeys have the smallest environmental footprint on the earth.**
   - **Beef and lamb have the largest environmental footprint on the earth.**
   - **Corn, Barley and Rye are carbohydrates sources with small environmental footprints.**

Environmental impact of pet food (an example):

The estimated cat population in the US is 74 million.

If all the cats in the US were to consume a high protein diet, they would have a large environmental footprint and occupy 158 million acres of land.

If the same population of cats were to consume a moderate protein diet, they would have a smaller environmental footprint and occupy 37 million acres of land.

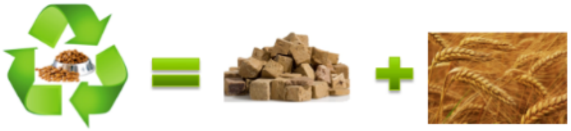

<u>**What is a nutritionally sustainable pet food?**</u>

1. Meets the nutrient requirements to promote health and vitality of our companion pets
2. Utilizes grains as a carbohydrate source
3. Utilizes environmentally friendly animal protein sources
4. Utilizes by-products to avoid direct competition with the human food chain

Because more than 12% of the 7.1 billion people in the world are undernourished …

☺ ☺ ☺ ☺ ☺ ☺ ☺ ⇨ ☹

(1 out of every 8)

It is important to consider the environmental impact not only of ourselves but also of our companion animals

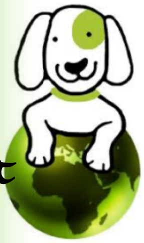

Supplement: Supplementary file 2 [file Image_2.PDF]
